# Supplementary material for: Effective Coverage of Maternal and Newborn Health Services in Sub-Saharan Africa: What distinguishes high from medium and low performers?
Source: PLoS One. 2026 Apr 13;21(4):e0347151. doi: 10.1371/journal.pone.0347151 (PMC13075690; doi:10.1371/journal.pone.0347151)
Supplement: S1 File — (DOCX) [file pone.0347151.s001.docx]

**Additional file 1**

**Estimation of effective coverage and health service coverage cascade of MNH visits**

Effective coverage is a composite measure of service coverage and is determined by combining contact coverage and intervention coverage rates. It is calculated using the formula ECij = ∑Qij×Uij|Nij = 1, where ECij represents the effective coverage for an individual i receiving intervention j. Here, Q denotes the proportion of potential health gain achieved through the intervention, which in this context equates to the average quality score of healthcare services received by women (1). U refers to the probability of receiving the intervention/utilization of services, conditional on need, and is represented as contact coverage rates for specific interventions/services such as attending 4^+^ ANC visits (2). For example, if contact coverage is 100%, it means all women in need of the service attended at least 4 ANC visits. Intervention coverage on the other hand reflects the quality of the services provided, indicating how well the services met the required standards. If the population average intervention coverage is 50%, then, EC will be 50%. This means that, on average, 50% of women who attended 4^+^ANC visits have received all the required services according to the standards.

In a scenario where contact coverage is 50% and intervention coverage is also 50%, the EC would be 25%. This indicates that 25% of women who needed the service both attended the visits and received all the required services, on average. When both contact and intervention coverage are not perfect, the EC will reflect the combined effect of these two factors, but the gap between coverage and EC will not always be halved. Instead, it will be proportional to both the intervention coverage score and the contact coverage rate. For instance, in Ethiopia's case, where contact coverage for 4^+^ANC visits is 33.34% and average intervention coverage is 21.1%, the EC becomes 7.03%. This means that only 7.03% of women both attended 4^+^ANC visits and received all the required services according to standards, on average. This example highlights the significant impact of low intervention coverage on overall EC. Even when access to services is high, low intervention coverage can drastically reduce the effective coverage, indicating the need to improve both the access to and the quality of services for better health outcomes. Therefore, EC of MNH services is the probability of having the needed health facility visits and receiving all the required services according to standards, on average.

The following tables show the key concepts of coverage cascades and list of quality service indicators about MNH services. Table1 shows the different levels of coverage cascade of routine MNH services used for the study. In our study, we used service coverage cascade, which covers as i) Service contact or contact coverage is the proportion of the target population who visit a health facility for perinatal services, ii) Intervention specific coverage is the proportion of the target population who receive the required health interventions (e.g., uptake of tetanus toxoid immunization in ANC visit), and iii) QAC is the proportion who receive service according to recommended standards of multiple dimensions of care. On the other hand, table 2 is used to show the list of indicators for the different domains of MNH services utilized.

**Table 1:** Service coverage cascade in the antenatal, childbirth and postnatal period.

| **MNH visits** | **Target populations** | **Service contact** | **Intervention specific coverage** | **EC or quality adjusted coverage (QAC)** |
| --- | --- | --- | --- | --- |
| Antenatal care visit | Women aged 15-49 years old who had given live birth in two years preceding the survey | Use at least four ANC visits from skilled providers | Use at 4ANC visits and receive key components ANC interventions | Received EC or QAC of 4ANC visits = average score of quality of 4ANC visits (Q)×proportion of 4ANC visits. |
| Institutional delivery | Women aged 15-49 years old who had given live birth in two years preceding the survey | Women give birth at HF among eligible women | Intrapartum care | Received EC or QAC of ID = average score of quality of ID (Q)×proportion of ID. |
| Postnatal care visit | Women aged 15-49 years old who had given live birth in two years preceding the survey | Mother and newborn receive PNC visit within 48 hours of childbirth | Receive PNC interventions | Received EC or QAC of PNC visit = average score of quality of PNC visits (Q)×proportion of PNC visit. |
| QAC= quality adjusted coverage; EC= effective coverage (%). Q= average quality score of all interventions (ranges 0 to 1, which is later changed to percentages for reporting). U= Utilization/contact coverage (range 0 to 100%). | | | | |

**Quality indicators of MNH services**

Intervention coverage was estimated based on the average quality score of MNH service indicators at the population level. Ten (10) items were utilised to estimate the average intervention coverage score for 4^+^ANC visits except for South Africa and Mauritania in which 9 items were used due to one indicator that is receipt of intestinal parasitic drugs during pregnancy being missing from the selected 10 indicators of service delivery quality in the dataset. In addition, 4 items for institutional delivery, 3 items for PNC for mothers within two days post-delivery and 8 items for PNC for newborns within two days post-delivery were considered to estimate service delivery quality for each woman and their newborns. Then, an overall average score was estimated for each of the domains at the population level. To do these, 0 value was given for women who did not visit health facilities and/or did not fulfil the required health facility visits according to the WHO standards. For those women who fulfilled the required visits, the average quality score was estimated based on the total number of indicator items, for each woman (**Table 2**).

**Table 2:** List of quality of MNH service indicator items.

| **A** | **Antenatal care interventions** |
| --- | --- |
| 1 | Intestinal parasitic drug/Albendazole taken during pregnancy (Not applicable for Mauritania and South Africa) |
| 2 | Iron taken during pregnancy |
| 3 | Iron taken at least 180 days |
| 4 | Blood pressure measured |
| 5 | Urine test done in pregnancy |
| 6 | Blood test done in pregnancy |
| 7 | Had ANC by skilled providers |
| 8 | Had two doses of TT in pregnancy |
| 9 | Had ANC at health facilities |
| 10 | Had first ANC within first 4 months |
| **B** | **Interventions of institutional delivery** |
| 1 | Stayed in hospital for 12 or more hours post-delivery |
| 2 | Delivery assisted by skilled providers |
| 3 | Skin to skin contact done immediately after birth and/or within 1 hour |
| 4 | Early initiation of breast feeding performed within 1hour |
| **C** | **Interventions of PNC for mothers** |
| 1 | PNC of mothers within two days |
| 2 | PNC of mother by trained health workers |
| 3 | PNC of mother at a health facility |
| **D** | **Interventions of PNC for newborns** |
| 1 | PNC of newborns within two days |
| 2 | PNC of newborns by trained health workers |
| 3 | PNC of newborns at health facility |
| 4 | Cord care examination performed |
| 5 | Body temperature measured |
| 6 | Counselling about newborn danger signs performed |
| 7 | Counselling on breastfeeding provided |
| 8 | Observed breastfeeding |

**Note**: Skilled and/or trained health workers for MNH services was estimated based on the following lists: Nurses, auxiliary nurse/ birth attendant and/or midwifes, physician assistants, and health officers for routine services as they are also tasked with midwifery skills. We have assumed that birth attendants/auxiliary nurse and/or midwifes are skilled as *routine care does not require specialized services unless complications arise.* However**,** we have not considered others, including health extension/community/village, and itinerant health workers.

**References**

1. Shengelia B, Tandon A, Adams OB, Murray CJL. Access, utilization, quality, and effective coverage: An integrated conceptual framework and measurement strategy. Social Science & Medicine. 2005;61(1):97-109.

2. Nguhiu PK, Barasa EW, Chuma J. Determining the effective coverage of maternal and child health services in Kenya, using demographic and health survey data sets: tracking progress towards universal health coverage. Trop Med Int Health. 2017;22(4):442-53.
