# Supplementary material for: Effective Coverage of Maternal and Newborn Health Services in Sub-Saharan Africa: What distinguishes high from medium and low performers?
Source: PLoS One. 2026 Apr 13;21(4):e0347151. doi: 10.1371/journal.pone.0347151 (PMC13075690; doi:10.1371/journal.pone.0347151)
Supplement: S2 File — (DOCX) [file pone.0347151.s002.docx]

**Additional file 2**

**Service Specific Availability and Readiness Indicators of MNH Services**

1. **Antenatal care service availability and readiness**

Availability is used to refer the percentage of health facilities in the countries health facility survey samples that reported offering specific services and the presence of tracer items for different inputs, such as the availability of diagnostic, essential medicines, and infrastructure resources across the health facilities in those countries. The structural quality in terms of availability of ANC services was assessed using four components of the service based on each country’s facility survey: Iron supplementation, tetanus toxoid vaccination, folic acid supplementation and monitoring for hypertensive disorders of pregnancy. However, these items are limited and may not provide a complete picture of the country’s capacity. Other additional indicators were extracted from Ghana and Liberia. However, those items were not found in the other countries health facility survey’s and are not utilized for comparison. In addition, the ANC service readiness of health facilities across the countries was assessed using 9 tracer items categorized in four domains of Trained Staff & guidelines, Equipment, Diagnostics, and Medicines & commodities. We did not found data about Trained Staff & guidelines in Kenya’s health facility surveys. Therefore, caution is needed in interpreting the overall capacity of countries for ANC service delivery.

**Table 1:** The percentage distribution of antenatal care service availability by country.

| **Country** | **Proportion of facilities offering antenatal care** | **Tracer indicators of ANC service availability** | | | | | | | | **Mean availability of tracer items** |
| --- | --- | --- | --- | --- | --- | --- | --- | --- | --- | --- |
|  |  | Iron supplementation | Folic acid supplementation | Tetanus toxoid vaccination | Monitoring for hypertensive disorder of pregnancy | Routine checks for urine protein | HIV testing for pregnant women | Routine syphilis testing for pregnant women | Treatment for pregnant women with syphilis |  |
| Ghana | 85% | 84% | 84% | 83% | 81% | 76% | 84% | 81% | 61% | 83% |
| Liberia | 89% | 86% | 78% | 84% | 85% | 37% | 80% | 44% | 63% | 84% |
| Kenya | 81% | 79% | 77% | 48% | 79% | N/A | N/A | N/A | N/A | 73% |
| Tanzania | 88% | 76% | 87% | 87% | 87% | N/A | N/A | N/A | N/A | 85% |
| Malawi | 60% | 59% | 49% | 59% | 57% | N/A | N/A | N/A | N/A | 57% |
| Ethiopia | 80% | 76% | 57% | 74% | 59% | N/A | N/A | N/A | N/A | 69% |

**Note:**

- Offers antenatal care refers to the proportion of health facilities in those countries that provide antenatal care services. The mean score is only for the four items.
- Mean availability is the overall average score of the mean availability scores of the four items in those health facilities.

**Table 2:** The percentage distribution of antenatal care service readiness tracer items by country.

| **Country** | **Service specific readiness score for ANC service delivery tracer items** | | | | | | | | | **Mean availability of tracer items** |
| --- | --- | --- | --- | --- | --- | --- | --- | --- | --- | --- |
|  | Guidelines available  antenatal care | ANC check lists and/or job aids | At least one trained staff antenatal care | Blood pressure Apparatus | Haemoglobin test | Urine dipstick protein test | Iron tablets | Folic acid tablets | Tetanus toxoid vaccine |  |
| Ghana | 43% | 60% | 60% | 94% | 52% | 73% | 67% | 85% | 67% | 67% |
| Liberia | 68% | 71% | 41% | 74% | 21% | 34% | 70% | 71% | 81% | 59% |
| Kenya | N/A | N/A | N/A | 95% | 31% | 45% | 86% | 88% | 97% | 74% |
| Tanzania | 49% | 53% | 49% | 99% | 65% | 57% | 92% | 94% | 91% | 72% |
| Malawi | 65% | 68% | 42% | 93% | 18% | 26% | 92% | 82% | 91% | 64% |
| Ethiopia | 29% | 57% | 44% | 69% | 8% | 15% | 66% | 60% | 35% | 43% |

**Table 3.** Health care financing, and Health worker, maternity bed and facility density scores across the selected SSA countries.

| **Countries** | Health worker density (Core health workers per 10, 000 population, Target 23) | Medical doctors (per 10,000) | Nursing and midwifery personnel (per 10,000) | Maternity beds density (Per 1,000, Target 10) | Facility density (Number of facilities per 10, 000, Target 2) | Average Health care financing per capital for the 2 years before the survey | Years of financing considered | Year of DHS |
| --- | --- | --- | --- | --- | --- | --- | --- | --- |
| Ghana | N/A | 1.642 (2020) | 34.956 (2020) | N/A | N/A | US$95.5 | 2020 & 2021 | 2022 |
| Liberia | 11(48%) | 0.503 (2018) | 19.256 (2018) | 11.1(100%) | 1.9 (95%) | US$71.5 | 2017&2018 | 2019-20 |
| Rwanda |  | 1.162 (2019) | 9.326 (2019) |  |  | US$49.5 | 2017&2018 | 2019-20 |
| Malawi | 10.4 (45%) | 0.31 (2016) | 2.954 (2016) | 8.7 (87%) | 0.6 (31%) | US$37.5 | 2013&2014 | 2015-16 |
| South Africa | N/A | 7.646 (2016) | 50.133 (2018) | N/A | N/A | US$515.5 | 2014&2015 | 2016 |
| Kenya | 15.6 (68) | 2.26 (2021) | 11.991 (2018) | 13.8 (138%) | 2.2 (110%) | US$90.5 | 2020&2021 | 2022 |
| Tanzania | 8.4 (36.5%) | 0.497 (2018) | 5.498 (2018) | N/A | 1.9 (95%) | US$35 | 2020&2021 | 2022 |
| Mali | N/A | 1.231 (2018) | 4.218 (2018) | N/A | N/A | US$28 | 2016&2017 | 2018 |
| Nigeria | N/A | 3.757 (2018) | 9.141 (2018) | N/A | N/A | US$73 | 2016&2017 | 2018 |
| Ethiopia | N/A | 0.755 (2018) | 7.013 (2108) | N/A | N/A | US$20.5 | 2013&2014 | 2015-16 |

**Note**: We used an average of the 2 years before the DHS survey of countries current health expenditure per capita data for comparison of the healthcare financing across countries. Source for healthcare financing: [Global Health Expenditure Database](https://apps.who.int/nha/database); for medical doctors (per 10,000 population) and Nursing and midwifery personnel (per 10,000 population): Global Health Observatory data repository. for others: countries health facility surveys. Core health workers include doctors, nurses and midwives.

1. **Institutional delivery and postnatal care service availability and readiness**

We have also explored the selected countries health facility surveys for service-specific availability and readiness capacity of institutional delivery and postnatal care. However, there is no clearly outlined data about routine delivery and postnatal care services. In addition, some countries surveys are published in French and we were unable to use it due to language limitations. We were also unable to get health facility surveys for some countries in the high, medium and low performing categories. This has forced us to consider other countries based on their relative performance rankings. Despite these limitations, we have found data for both basic and comprehensive emergency obstetric and newborn care (BEmONC) services of the included countries. In our analysis, we used BEmONC service scores as a proxy measure of country capacity to provide routine institutional delivery and PNC services. Accordingly, service specific availability for institutional delivery and PNC was assessed using an average score of selected BEmONC service delivery indicator tracer items. Readiness, on the other hand, is a composite measure calculated for the facilities that provide the service (restricted to the subset of facilities that offered the specific service). The components of the readiness score vary depending on the service but generally include domains such as key staff members with essential trainings, equipment, medicines and supplies, and diagnostics. A readiness score of 50 indicates that, on average, half of the facilities offering the service had all the necessary inputs for service delivery (**Table 4** and **5**).

**Table 4:** The percentage distribution of Basic Emergency and Essential Obstetric and Newborn Care services availability by country.

| **Country** | **Offers delivery services** | Parenteral administration of antibiotics | Parenteral administration of oxytocic drugs | Parenteral administration of anticonvulsants | Assisted vaginal delivery | Manual removal of placenta | Manual removal of retained products | **Mean availability of obstetric signal functions offered** | Antibiotics for preterm or prolonged PROM | Neonatal resuscitation | Corticosteroids in preterm labour | KMC for premature/very small babies | Injectable antibiotics for neonatal sepsis | **Mean availability of newborn signal functions offered** | Administration of oxytocin for the prevention of post-partum haemorrhage | Monitoring and management of labour using partograph | Immediate and exclusive breastfeeding | Hygienic cord care | Thermal protection | **Mean availability of tracer items for routine perinatal care** | **Overall mean availability score of BEm/EONC tracer items** |
| --- | --- | --- | --- | --- | --- | --- | --- | --- | --- | --- | --- | --- | --- | --- | --- | --- | --- | --- | --- | --- | --- |
| Rwanda | N/A | N/A | N/A | N/A | N/A | N/A | N/A | **N/A** | N/A | N/A | N/A | N/A | N/A | **N/A** | N/A | N/A | N/A | N/A | N/A | **N/A** | **N/A** |
| Malawi | 51% | 96% | 98% | 85% | 71% | 79% | 60% | **82%** | N/A | 97% | N/A | N/A | N/A | **N/A** | N/A | N/A | N/A | N/A | N/A | **N/A** | **N/A** |
| South Africa | N/A | N/A | N/A | N/A | N/A | N/A | N/A | **N/A** | N/A | N/A | N/A | N/A | N/A | **N/A** | N/A | N/A | N/A | N/A | N/A | **N/A** | **N/A** |
| Ghana | 71% | 80% | 96% | 66% | 31% | 89% | 47% | **68%** | 67% | 88% | 31% | 15% | 70% | **54%** | 99% | 92% | 100% | 99% | 99% | **98%** | **73%** |
| Kenya | 50% | 81% | 87% | 54% | 22% | 66% | 42% | **59%** | 76% | 60% | 48% | 47% | 62% | **59%** | 99% | 85% | 98% | 92% | 95% | **94%** | **70%** |
| Tanzania | 71% | 53% | 53% | 51% | 53% | 52% | 54% | **53%** | 42% | 57% | 25% | 24% | 40% | **38%** | 70% | 68% | 70% | 70% | 70% | **70%** | **53%** |
| Nigeria | N/A | N/A | N/A | N/A | N/A | N/A | N/A | **N/A** | N/A | N/A | N/A | N/A | N/A | **N/A** | N/A | N/A | N/A | N/A | N/A | **N/A** | **N/A** |
| Ethiopia | 55% | 46% | 51% | 28% | 39% | 55% | 53% | **45%** | 39% | 54% | 9% | 46% | 31% | **36%** | 55% | 48% | 55% | 26% | 53% | **47%** | **43%** |

**Note:** N/A is used to indicate non-availability of data due to absence of country health facility surveys that reported the items included indicators. If there is a survey, either it does not go in line with the timing of their DHS surveys and/or do not provide data according to the set of the included indicators for MNH service delivery. In addition, mean availability does not consider the proportion of health facilities.

**Table 5:** The percentage distribution of Basic Emergency and Essential Obstetric and Newborn Care services readiness by Country.

| Country | Guidelines for essential childbirth care | Check-lists and/or job-aids for essential childbirth care | Guidelines for essential newborn care | At least one staff trained in essential childbirth care | Staff trained in newborn resuscitation | Emergency transport | Sterilization equipment | Examination light | Delivery pack | Suction apparatus | Manual vacuum extractor | Vacuum aspirator or D&C kit | Neonatal bag and mask | Delivery bed | Partograph | Gloves | Infant weighting scale | Blood pressure apparatus | Soap and running water OR alcohol-based hand rub | Antibiotic eye ointment | Injectable uterotonic | Injectable antibiotic | Magnesium sulphate (injectable) | Skin disinfectant | Intravenous solution with infusion set | **Mean readiness score of tracer items** |
| --- | --- | --- | --- | --- | --- | --- | --- | --- | --- | --- | --- | --- | --- | --- | --- | --- | --- | --- | --- | --- | --- | --- | --- | --- | --- | --- |
| Rwanda | N/A | N/A | N/A | N/A | N/A | N/A | N/A | N/A | N/A | N/A | N/A | N/A | N/A | N/A | N/A | N/A | N/A | N/A | N/A | N/A | N/A | N/A | N/A | N/A | N/A | **N/A** |
| Malawi | 61% | 64% | **N/A** | 40% | 63% | 82% | 47% | 58% | 55% | 80% | 47% | 37% | N/A | 55% | **N/A** | 96% | 84% | 92% | 71% | 85% | 98% | 92% | 90% | 88% | 62% | **70%** |
| South Africa | N/A | N/A | N/A | N/A | N/A | N/A | N/A | N/A | N/A | N/A | N/A | N/A | N/A | N/A | N/A | N/A | N/A | N/A | N/A | N/A | N/A | N/A | N/A | N/A | N/A | **N/A** |
| Ghana | 31% | 54% | 29% | 45% | 52% | 37% | 51% | 58% | 91% | 69% | 72% | 39% | 52% | 82% | 86% | 97% | 92% | 94% | N/A | 55% | 94% | 70% | 65% | 55% | 90% | **65%** |
| Kenya |  |  |  |  |  |  | 55% | 48% | 80% | 58% | 27% | 42% | 38% | 91% | 74% | 92% | 67% | 76% | 74% | 89% | 87% | 73% | 54% | 83% | 82% | **68%** |
| Tanzania | 46% | 59% | 40% | 31% | 29% | 15% | 66% | 41**%** | 95% | 85% | 24% | 26% | 51% | 93% | 87% | 97% | 89% | 96% | 96% | 77% | 97% | 96% | 95% | 96% | 96% | **69%** |
| Nigeria | N/A | N/A | N/A | N/A | N/A | N/A | N/A | N/A | N/A | N/A | N/A | N/A | N/A | N/A | N/A | N/A | N/A | N/A | N/A | N/A | N/A | N/A | N/A | N/A | N/A | **N/A** |
| Ethiopia | 26% | 50% | 30% | 33% | 51% | 84% | 66% | 53**%** | 98% | 72% | 54% | 37% | 73% | 100% | 80% | 95% | 95% | 85% | 56% | 92% | 83% | 84% | 36% | 94% | 83% | **68%** |

**Community and/or societal level factors**

Disparities in community and/or societal-level factors such as education, household size, media access, and political stability can also play a significant role in determining variations in EC of MNH services even when healthcare service availability and readiness scores are relatively similar across countries. According to The GlobalEconomy.com, the political stability index is a composite measure derived from multiple sources, including the Economist Intelligence Unit, the World Economic Forum, and Political Risk Services. It assesses the likelihood of disruptions such as undemocratic transfers of power, armed conflicts, violent protests, social unrest, international disputes, terrorism, and ethnic or regional tensions. The index, available from 1996 to 2023, is measured on a continuum scale (-2.5 weak; 2.5 strong), where negative values indicate weak political stability and positive values signify strong stability. The consistency of the index over time allows for meaningful comparisons across different periods and countries **(Table 6)**.

**Tabel 6:** Distribution of community/societal level factors across the selected countries DHS and The GlobalEconomy.com Data.

| **Country** | Proportion of women with some or more school attendance (%) | Proportion of women with primary education (%) | Proportion of women with sone secondary or higher education (%) | Proportion of women employed in the 12 months before surveys (%) | Proportion of women in the middle or more wealth quintiles (%) | Family size/Total fertility rate (average score) | Household size (average score) | Proportion of women with internet access (%) | Proportion of women with mass media access (%) | Average political stability index 1996 to 2023 (-2.5 weak; 2.5 strong) |
| --- | --- | --- | --- | --- | --- | --- | --- | --- | --- | --- |
| Ghana | 83.9 | 13.8 | 70.1 | 78.2 | 65.6 | 3.9 | 3.5 | 43.3 | 72.7 | -0.02 |
| Liberia | 69.3 | 23.7 | 45.6 | 64.3 | 65.2 | 4.2 | 4.6 | 22.0 | 33.1 | -0.95 |
| Rwanda | 90.6 | 58.3 | 32.3 | 73.3 | 62.4 | 4.1 | 4.3 | 12.3 | 65.6 | -0.52 |
| Malawi | 87.9 | 62.1 | 25.8 | 67.1 | 61.7 | 4.4 | 4.5 | 5.5 | 37.2 | -0.09 |
| South Africa | 98 | 9.1 | 88.9 | 38.5 | 60.5 | 2.6 | 3.4 | 47.4 | 82.3 | -0.22 |
| Kenya | 94.5 | 36.3 | 58.1 | 59.7 | 66.7 | 3.4 | 3.7 | 44.2 | 78.5 | -1.15 |
| Tanzania | 83.9 | 53.2 | 30.7 | 64.3 | 66.9 | 4.8 | 4.5 | 12.8 | 45.6 | -0.37 |
| Mali | 34 | 13 | 21 | 61 | N/A | 6.3 | 5.8 | N/A | N/A | -0.90 |
| Nigeria | 65.1 | 14.4 | 50.7 | 68.4 | 63.5 | 5.3 | 4.7 | 15.7 | 44.4 | -1.81 |
| Ethiopia | 52.2 | 35 | 17.2 | 50.2 | 65.3 | 4.6 | 4.6 | 4.4 | 26.4 | -1.52 |

**Note:** Data are collected from countries health facility, DHS, TheGlobalEconomic.com, [Global Health Expenditure Database](https://apps.who.int/nha/database) and Global Health Observatory data repository. The three databases were used to collect health care financing, political stability index and core health workers density for the included countries, respectively.
